# Oropharyngeal Dysphagia After Hospitalization for COVID-19 Disease: Our Screening Results

**DOI:** 10.1007/s00455-021-10325-0

**Published:** 2021-06-24

**Authors:** Maria Raffaella Marchese, Carolina Ausili Cefaro, Giorgia Mari, Ilaria Proietti, Angelo Carfì, Matteo Tosato, Ylenia Longobardi, Lucia D’Alatri, “Gemelli Against COVID-19 Post-Acute Care Team”

**Affiliations:** 1 Department of Head and Neck Sciences, Catholic University of Sacred Heart, Policlinico “A. Gemelli” Foundation, L.go “A. Gemelli” 8, 00168 Rome, Italy; 2 Division of Phonatrics, Department of Aging, Neuroscience, Orthopedics and Head and Neck Sciences, Fondazione Policlinico Universitario A. Gemelli IRCCS, Rome, Italy; 3 Institute of Internal Medicine and Geriatrics, Università Cattolica del Sacro Cuore, Rome, Italy; 4 Institute of Otorhinolaryngology, Università Cattolica del Sacro Cuore, Rome, Italy

**Keywords:** SARS-CoV-2, COVID-19, Dysphagia, Oropharyngeal dysphagia, Post-COVID-19

## Abstract

A high percentage of patients suffered symptoms also after recovery from the Coronavirus Disease—2019 (COVID-19) infection. It is not well clear what are the specific long-term sequelae (complications and symptoms). During the acute phase the patients may develop a multi-organ system pathology including aerodigestive tract. As the pathophysiology of COVID-19 emerges, the aim of our study was to describe the prevalence of oropharyngeal dysphagia after COVID-19 disease. From March to July 2020 we enrolled patients recovered from SARS-CoV-2 infection who had been previously hospitalized for the disease. They were screened for dysphagia by mean of the Eating Assessment Tool-10 (EAT-10). The cases with EAT-10 score > 3 were graded for the aspiration risk by applying the Gugging Swallowing Screen (GUSS) and were submitted to the Swal-QoL questionnaire. The cases with a GUSS score > 19 were subjected to FEES. 8/117 (7%) patients had positive screening result. 4/8 (50%) revealed an abnormal health related quality of life in oropharyngeal dysphagia with a mean Swal-QoL score of 69.73. The most affected domain was the “time of meals” (mean score 65) following by the “sleep” (mean score 66) and “eating desire” (mean score 72). 1/8 cases showed increased risk for aspiration and did not showed endoscopic signs of oropharyngeal dysphagia. Our results showed that the prevalence of upper dysphagia after hospitalization for SARS-CoV-2 is not anecdotal and that probably this long-lasting sequela has a psychogenic etiology.

## Introduction

Coronavirus disease (COVID-19) is a viral respiratory illness [1]. Whereas 80–90% of infected individuals are asymptomatic or experience mild symptoms like fever, dry cough, and fatigue, the minority of cases develop a respiratory distress or respiratory failure and hence the need for intensive care unit (ICU) [1]. Specifically Abate et al. [2] reported that more than one-third of patients with coronavirus infection were admitted to ICU globally. The severity of the disease is related to age and comorbidities [3] and influences the duration of manifestations. For mild cases the symptoms may last for 2 weeks while for the severe ones until 6 weeks [4]. The recovery from disease could be confirmed by PCR or by the absence of the symptoms for several days. Similarly to post-acute viral syndromes described in survivors of other virulent coronavirus epidemics, there are increasing reports of persistent and prolonged effects after acute COVID-19 [5–7]. The most recent studies reported an incidence of post-acute infectious consequences of COVID-19 ranging from 32.6 [6] to 87.4% [8] at 60 days follow-up. It is not yet well clear what are the specific long-term sequelae (complications and symptoms) but it seems they mostly differ from the typical manifestations of the COVID-19 disease [9, 10]. As described by Nalbandian (2021) et al. [11] they varied from a low-critical symptoms like headache, anxiety, joint pain or fatigue to more critical conditions such as myocarditis, renal failure and pulmonary fibrosis that may be one or associated. Acute COVID-19 usually lasts until 4 weeks from the onset of symptoms, beyond which replication-competent SARS-CoV-2 has not been isolated [11]. While the definition of the post-acute COVID-19 timeline is evolving, it has been suggested to include persistence of symptoms or development of sequelae beyond 4–12 weeks from the onset of acute symptoms of COVID-19 [12, 13], as replication-competent SARS-CoV-2 has not been isolated after 3 weeks [14].

The relationship between post-extubation and dysphagia is well-known, as well as that patients who have had oral endotracheal intubation with mechanical ventilation may be at risk for swallowing disorders [15–18]. Nevertheless, there are also factors specifically related to the COVID-19 disease that could contribute to the occurrence of oropharyngeal dysphagia (OPD): mean old age (> 65 years) [19], cognitive deterioration and muscular weakness related to the prolonged hospitalization in ICU [20], neurologic symptoms SARS-CoV-2 related [21] and poor quality of life or stress during quarantine [22]. Moreover Aoyagi et al. [23] described a case of oropharyngeal dysphagia likely due to the involvement of glossopharyngeal and vagal nerves following SARS-CoV-2 infection. Particularly the OPD is strongly associated with community acquired pneumonia and should be considered as an independent risk factor of this last [24]. Therefore the OPD with aspiration could represent an additional risk to develop pneumonia in SARS-CoV-2 patients.

Aim of our study was to describe the prevalence, severity and features of OPD after hospitalization and recovery from the COVID-19 disease.

## Materials and Methods

**Fig. 1:**
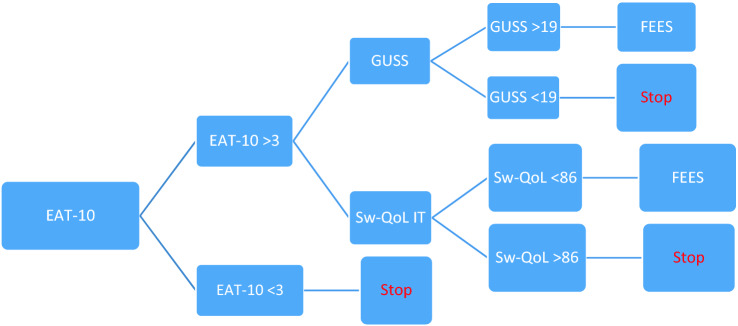
Flow-chart of the dysphagia assessment protocol

We enrolled subjects who were going to the Post-COVID Day Hospital instituted by the Fondazione Policlinico A. Gemelli IRCCS in Rome for the purpose to offer a multidisciplinary assistance after the disease. We selected the patients from March to July 2020 basing on the following inclusion criteria: double negative nucleic acid SARS-CoV-2 test on nasopharyngeal and oropharyngeal specimens, age > 18 and < 80 years, BMI < 30, past hospitalization because of COVID-19 disease. The exclusion criteria were: history of dysphagia, neurologic and psychiatric disease, mental deficit and of oral endotracheal intubation before SARS-CoV-2 hospitalization; previous larynx, neck, esophagus, stomach or lung surgery or disease, immunomediated disease. All cases were submitted upon admission to the Eat Assessment Tool-10 (EAT-10) [25]. Patients with the EAT score ≥ 3, within  1 week from the screening test, underwent assessment of the severity of aspiration risk by mean of Gugging Swallowing Screen (GUSS) [26] and assessment of the health related quality of life in oropharyngeal dysphagia by the Swallowing Quality of Life (Swal-QoL—Italian version) questionnaire [27]. Both tests were administered by an expert speech pathologist. Patients with a GUSS score > 19 and/or with a Swal-QoL score < 86 were referred to Fiberoptic Endoscopic Evaluation of Swallowing (FEES) which findings were scored basing on the Pooling-score (dysphagia if score < 5) [28] (Fig. 1). Moreover, for each patient the following data were recorded: age, gender, BMI, date of onset symptoms, overall hospitalization length, duration of ICU hospitalization, type of ventilation and duration of intubation. A written informed consent was obtained from all the participants included in the study.

Data analysis was performed using commercially available software (Excel–Microsoft Corporation, Redmond, Washington, USA). Continuously distributed data were summarized as the mean and categorical variables with frequencies and percentages.

## Results

From March to July 2020 we enrolled 117 patients admitted to the post-COVID Day Hospital of Policlinico A.Gemelli IRCCS in Rome. All patients (117/117 – 100%) were elegible for our study. The female were 48/117 (41%) and the males 69/117 (59%) with a mean age of 57 years (range 43–71 years) and a mean BMI of 29.14 (range 24–32). At the time of hospital admission the symptom was dyspnea in 68/117 (58%) and cough in 58/117 (49%). The mean length of hospitalization was 17.6 days (range 7–28). 106/117 (90%) underwent supplemental oxygen and 10/117 (8.5%) oral endotracheal intubation.

The EAT-10 was administered on average 82 days (range 8–122 days) after the symptoms onset and a mean of 73 days (range 30–104 days) after the hospital admission. The mean score of EAT-10 was 0.9 (range 0–7). The number of patients with a positive screening test were 8/117 (7%) (EAT-10+) and 109/117 (93%) the negative ones (EAT-10−). The mean score of the group EAT-10+ was 5.8 (range 4–7) and the mean score in the group EAT-10− was 1.6 (range 0–2). The demographic, antropometric, onset symptoms and hospitalization related data of both EAT-10+ and EAT-10− are showed in the Table 1.

**Table 1:** Epidemiologic, antropometric and clinical data of EAT-10+ and EAT-10− groups

	EAT-10 > 3	EAT-10 < 3
Number of patients	8/117 (6.83%)	109/117 (93.16%)
Females	6/8 (75%)	42/117 (39%)
Males	2/8 (25%)	67/117 (61%)
Mean age (years)	54 (± 19.23)	56.7 (± 13.93)
BMI	25.56 (± 6.67)	29.18 (± 4.71)
Duration of hospitalisation (days)	16.5 (± 14)	17.7 (± 10.7)
Oxygen supplement (n. patients)	4/8 (50%)	102/109 (94%)
Oral endotracheal intubation (n. patients)	3/8 (38%)	7/109 (6%)
Presenting symptoms		
*Dyspnea*	7/8 (87.5%)	68/117 (58%)
*Cough*	5/8 (62.5%)	58/117 (49%)

The Swal-QoL questionnaire was administered to all cases of the EAT-10+ group (8/8 – 100%). The mean score was 80.18 (range 53.91–94.67). 4/8 (50%) revealed an abnormal health related quality of life in oropharyngeal dysphagia with a mean score of 69.73 (range 53.91–81.82). The most affected domain was the “*time of meals*” (mean score 65) following by the “*sleep*” (mean score 66) and “*eating desire*” (mean score 72). The mean scores obtained for each domains are showed in decreasing order in the Table 2. The most recurrent abnormal (< 86) domains were, with the same result, “*sleep*”, “*mental health*”, “*fatigue*” and “*eating desire*” (5/8 subjects – 62.5%).

**Table 2:** The mean score of each domain of the Swal-QoL questionnaire

Domains of Swal-QoL questionnaire	Mean score
Time of meals	65
Sleep	66
Fatigue	72
Desire of meals	72
Depression	74
Overall impact	81
Symptoms	82
Fears	89
Selection of foods	92
Communication	92
Social impact	95

The mean score of each item and the recurrence of the answer for each item with a score lower than 5 (corresponding to a frequency of situation’s occurrence “almost never”, “sometimes”, “almost always” or “always”) are showed in increasing order in the Table 3. The item with the lowest score was “*I have sleep problems*” (mean score = 2).

**Table 3:** The list of items for each domain of the Swal-QoL questionnaire, in decreasing order basing on the prevalence of the answers different from “never” in the EAT-10+ group, and the related mean score

	ITEMS of the Swal-QoL questionnaire	% of answers different from “Never”	Mean score
Overall impact			
1	Dealing with my swallowing problems is very difficult	5/8 (62.5%)	4
2	My swallowing problems is a major distraction in my life	3/8 (37.5%)	4
	*Desire of meals*		
5	I’m rarely hungry anymore	6/8 (75%)	4
3	Most days, I don’t care if I eat or not	4/8 (50%)	4
4	I don’t enjoy eating anymore	3/8 (37.5%)	4
Time of meals			
6	My meal lasts a long time	5/8 (62.5%)	3
7	I takes me longer to eat than other people	4/8 (50%)	3
Symptoms			
10	Choking when you take liquids	6/8 (75%)	4
13	Having to clear your throat	6/8 (75%)	4
17	Food sticking in your throat	6/8 (75%)	4
9	Choking when you eat food	5/8 (62.5%)	4
16	Raclage	5/8 (62.5%)	3
8	Coughing	4/8 (50%)	4
15	Having excess saliva or phlegm	4/8 (50%)	4
11	Having thick saliva or phlegm	3/8 (37.5%)	4
12	Gagging	3/8 (37.5%)	4
14	Problems chewing	3/8 (37.5%)	4
18	Food sticking in your mouth	1/8 (12.5%)	4
19	Food or liquid dribbling out of your mouth	1/8 (12.5%)	5
21	I am not able to cough when food gets stuck	1/8 (12.5%)	5
20	Food or liquid coming out your nose	0/8 (0%)	5
Selection of foods			
22	The choice of the food is difficult	1/8 (12.5%)	5
23	It is difficult to find foods that I both like and can eat	1/8 (12.5%)	5
Communication			
24	People have hard time understanding me	2/8 (25%)	5
25	It’s difficult for me to speak clearly	2/8 (25%)	5
Fears			
27	I worry about getting pneumonia	3/8 (37.5%)	4
26	I am afraid of choking when I eat food	2/8 (25%)	4
29	I never know I am going to choke	2/8 (25%)	4
28	I am afraid of choking when I drink liquids	1/8 (12.5%)	5
Depression			
32	My swallowing problem frustrates me	5/8 (62.5%)	4
30	My swallowing problem depresses me	4/8 (50%)	4
31	My swallowing problem embarrasses me	4/8 (50%)	4
33	My swallowing problem bothers me	4/8 (50%)	3
34	I get impatient dealing with my swallowing problem	4/8 (50%)	3
Social impact			
35	I do not go out because of my swallowing problem	2/8 (25%)	4
36	My swallowing problem makes it hard to have a social life	2/8 (25%)	5
38	Social gatherings are not enjoyable because of my swallowing problem	1/8 (12.5%)	5
37	My usual work or leisure activities have changed because of my swallowing problem	0/8 (0%)	5
39	My role with my family and friends has changed because of my swallowing problem	0/8 (0%)	5
Fatigue			
40	I feel weak	6/8 (75%)	3
41	I feel fatigued	6/8 (75%)	3
42	I feel exhausted	6/8 (62.5%)	4
Sleep			
44	I have sleep problems	7/8 (87.5%)	2
43	I wake up at night	4/8 (50%)	3

8/8 (100%) cases with EAT-10+ underwent assessment of the severity of aspiration risk (GUSS) with a mean score of 18.3 (range 16–21). 7/8 (87.5%) subjects did not reach the cut-off for aspiration risk (mean score 17.9, range 16–19). The remaining case 1/8 (12.5%) (score = 21) was subjected to the flexible endoscopic examination of swallowing and did not showed signs of dysphagia (Pooling-score < 5).

## Discussion

Although COVID-19 is seen as a disease that primarily affects the lungs, it can damage many other organs as well. This organ damage may increase the risk of long-term health problems. Older people and people with many serious medical conditions are the most likely to experience lingering COVID-19 symptoms, but even young, otherwise healthy people can feel unwell for weeks to months after infection [7]. These people sometimes describe themselves as "long haulers" and the condition has been called post-COVID-19 syndrome or "long COVID-19” [1]. We first described the prevalence of dysphagia in discharged patients after hospitalization for COVID-19 disease. Even if the prevalence (7%) in our screened group of patients was small and did not require therapeutic intervention, it was not negligible. Our research came from the consideration that overall the patient affected by SARS-CoV-2 is exposed to a higher probability of dysphagia onset because of specific and non-specific risk factors. At the first wave of the pandemia the typical SARS-CoV-2-affected patients over 65 years old who developed acute breathing problems and in some cases were subjected to oral endotracheal intubation and long-term and isolated hospitalization in ICU or in the ward. Furthermore, the type of pneumonia often associated with COVID-19 can cause long-standing damage to the tiny air sacs (alveoli) in the lungs. The resulting scar tissue can lead to long-term breathing problems predisposing to abnormal coordination of breathing and swallowing.

Moreover the COVID19-affected patient was exposed to specific risks for develop swallowing abnormalities. As described by Aoyagi et al. [10] the SARS-CoV-2 infection may cause peripheric neuropathy of the IX and X cranial nerves. Moreover Ishkanian et al. [29] reported a case of dysphagia related to myositis caused by the virus. It is well known that aspiration, pneumonia, malnutrition, increased mortality, prolonged hospitalization, advanced disability and declining quality of life may be the consequence of oropharyngeal dysphagia and consequently it is not difficult to image how dangerous the effect of an hypothetical overlap between dysphagia and SARS-CoV-2 infection would be.

As reported by Dawson et al. [30] almost 30% of COVID-affected patients admitted in either intensive care unit or in the ward with respiratory issues were referred for swallow assessment.

Anyway, dysphagia, its management and the hypothesized causes as a result of COVID-19 remains to be fully defined. No prospective studies have explored this specific lasting sequela. Dysphagia in post-acute COVID-19 infections derives probably from a multiplicity of causes. In our group of subjects the dysphagia was on average described as difficulty to swallow liquids, stuck in the throat and raclage. This functional abnormality impacted on the time of meals and particularly on the sleep quality, mental health and perception of fatigue. Nevertheless only one case showed increased risk of aspiration even if without endoscopic findings of abnormal oropharyngeal swallowing. Anyway, dysphagia after hospitalization for COVID-19 disease was not anecdotal and it was long-lasting and independent of the oral endotracheal intubation.

Dawson et al. [30] found that dysphagia co-existed with delirium, fatigue and difficulty achieving effective breathe/swallow co-ordination. It is known that the psychosocial impact of COVID-19 is significant because mainly related to double stressors: quarantine and/or hospitalization. Brooks et al. [31] suggested that the psychosocial quarantine’s impact is wide-ranging, substantial and can be long lasting. They identified stress sources during quarantine (duration, fears of infection, frustration and boredom, inadequate supplies, inadequate information) and after quarantine (finances, stigma). Moreover Bo et al. [32] and Zhang et al. [33] reported a very high prevalence (96.2%) of post-traumatic stress disorder and depression (29.2%) in hospitalized COVID-19-affected patients. Based on our findings and in the light of these literature data we cannot exclude a psychogenic etiology of the dysphagia.

At the time of writing, the most part of papers about dysphagia in COVID-19 disease are expert consensus publications focused to provide practice guidelines to perform swallowing assessment minimizing the risk of infection [34]. Dawson et al. [30] performed a “face-to-face” swallow and voice quality assessment. Particularly they performed the cranial nerve and oro-motor examination of intra-oral musculature. As reported by Kimura et al. [34] in the current pandemic context, the clinical swallowing assessment without producing aerosols is more preferable compare to the Aerosol-Generating Procedures (AGPs). For this reason the employ of dysphagia screening tools such as the EAT-10 are strongly encouraged. On the other hand flexible endoscopic evaluation of swallowing is considered an incredible high risk AGP. In compliance of these considerations, we designed a multilevel assessment protocol starting just from the EAT-10 to select the cases worthy of graded dysphagia and risk of aspiration evaluation by the GUSS and the Swal-QoL questionnaire in turn to perform the FEES only after two levels of selection.

In conclusion our study, that fully meets the researchers’ recommendation to closely monitor the people who have had COVID-19 to see how their organs are functioning after recovery [35], contributes on the one hand to increase the still few evidences about the swallowing abnormalities in SARS-CoV-2 patients, particularly in the post-infection phase, and on the other it suggests a safety protocol for the swallowing assessment.

However, trials with a higher number of hospitalized cases are needed to confirm these findings. As above mentioned, the definition of the post-acute COVID-19 timeline is evolving, therefore further investigations focused on OPD extended from the acute phase to the post-infection phase and for a longer time after admission could help to define more accurately the prevalence of the OPD and to better understand its etiology. Finally, because it cannot be excluded that some of the possible long-term effects can affect also patients who suffered mild manifestations of COVID-19 infection, in the future might be interesting to include in the dysphagia screening all the categories of disease severity. Overall, in the light of the last research purposes, we believe that to standardize a secure protocol of OPD assessment that minimize the risk to spread the infection is paramount.

## Data Availability

The Authors have full control of all primary data and they are agree to allow the journal to review their data if requested.
